# Phenotypic and genotypic characteristics of *Escherichia coli* with non-susceptibility to quinolones isolated from environmental samples on pig farms

**DOI:** 10.1186/s40813-019-0116-y

**Published:** 2019-02-28

**Authors:** Patrick Kindle, Katrin Zurfluh, Magdalena Nüesch-Inderbinen, Sereina von Ah, Xaver Sidler, Roger Stephan, Dolf Kümmerlen

**Affiliations:** 1 Vetsuisse Faculty, Institute for Food Safety and Hygiene, University of Zurich, Winterthurerstrasse 272, CH-8057 Zurich, Switzerland; 2 Department of Farm Animals, Division of Swine Medicine, Vetsuisse Faculty, University of Zurich, Winterthurerstrasse 260, 8057 Zurich, Switzerland

**Keywords:** Antimicrobial resistance, Fluoroquinolones, Pigs, *Escherichia coli*, Genotypes

## Abstract

**Background:**

In the last decade, the growth of the pig-farming industry has led to an increase in antibiotic use, including several used in human medicine, e.g. (fluoro)quinolones. Data from several studies suggest that there is a link between the agricultural use of antibiotics and the prevalence of antibiotic-resistant bacteria in the pig farm environment, including (fluoro)quinolone resistance. This poses a threat to human and animal health. Our goal was to phenotypically and genotypically characterize 174 *E. coli* showing non-susceptibility to quinolones isolated from environmental samples from pig farms. Antimicrobial susceptibility testing (AST) was performed using the disk diffusion method. PCR and sequence analysis were performed to identify chromosomal mutations in the quinolone resistance-determining regions (QRDR) of *gyrA* and the isolates were screened for the presence of the plasmid-mediated quinolone resistance (PMQR) genes *aac-(6')-Ib-cr*, *qepA*, *qnrA*, *qnrB*, *qnrC*, *qnrD* and *qnrS*.

Strain relatedness was assessed by phylogenetic classification and multilocus sequence typing (MLST).

**Results:**

Of 174 isolates, 81% (*n* = 141) were resistant to nalidixic acid, and 19% (*n* = 33) were intermediately resistant. Overall, 68.4% (*n* = 119) were multidrug resistant. This study revealed a prevalence of 79.9% (*n* = 139) for *gyrA* QRDR mutations, and detected 21.8% (*n* = 38) isolates with at least one PMQR gene. The two most frequently detected PMQR genes were *qnrB* and *qnrS* (13.8% (*n* = 24) and 9.8% (*n* = 17, respectively). *E. coli* belonging to phylogenetic group A (48.3%/*n* = 84) and group B1 (33.3% /*n* = 58) were the most frequent. *E. coli* ST10 (*n* = 20) and ST297 (n = 20) were the most common STs.

**Conclusions:**

*E. coli* with non-susceptibility to quinolones are widespread among the environment of Swiss pig farms and are often associated with an MDR phenotype. In several cases these isolates possess at least one PMQR gene, which could spread by horizontal gene transfer. *E. coli* from pig farms have diverse STs, some of which are associated with human and animal disease.

**Electronic supplementary material:**

The online version of this article (10.1186/s40813-019-0116-y) contains supplementary material, which is available to authorized users.

## Introduction

Bacterial diseases in pigs reared on production farms are responsible for high morbidity and mortality rates and subsequently also for increased economic losses [1, 2]. The main indications for antibiotic therapy in pigs in Switzerland are gastrointestinal and respiratory diseases [3]. Therapy may include (fluoro)quinolones, which are categorized by the World Health Organization (WHO) as critically important antimicrobial agents (CIAs) [4]. Despite the worldwide growth of the pig-farming industry, monitoring systems on the use of antibiotics in veterinary medicine have led to reduced antibiotic use in many European countries, including Germany, Denmark, The Netherlands, and Sweden [5].

In pig husbandry, the usual way of drug application is oral, via the feed mixture. This is a practical way of drug application from a farmer’s point of view, but the drawback is the release of antimicrobial substances into the farm environment [6]. Data from several studies suggest that there is a link between the agricultural use of antibiotics and the prevalence of antibiotic-resistant bacteria in the pig farm environment, including (fluoro)quinolone resistance [7–11].

The pathways of antibiotic residues into the environment after animal treatment are numerous. Moreover, certain antibiotics such as fluoroquinolones and tetracyclines are not fully metabolized in pigs and their residues may be detected in dust, manure, sewage, soil, ground- and surface water and crops [6, 12–15]. These various antibiotic residue reservoirs are perfect breeding grounds for resistant bacteria, including (fluoro)quinolone resistant *E. coli* [16]. During a previous study aimed at analysing the use of fluoroquinolones in Swiss pig farms (von Ah, et al., manuscript under review), quinolone non-susceptible *E. coli* were isolated from environmental samples (dust, liquid manure and wipe samples of bay walls) of the farms. The goal of this study was to characterize these isolates with regard to the two main mechanisms of (fluoro)quinolone resistance in Enterobacteriaceae, i.e., the accumulation of mutations in the Quinolone Resistance Determining Region (QRDR) of *gyrA* encoding DNA gyrase, and the acquisition of plasmid-mediated quinolone resistance (PMQR) genes [11]. A further aim was to characterize the strains by phylogenetic grouping and multilocus sequence typing (MLST).

## Material and methods

### Strains and data collection

In this study, we analysed 174 *E. coli* isolates collected during 2016 by the Division of Swine Medicine of the Vetsuisse Faculty Zurich (von Ah, et al., manuscript under review). The collection consisted of strains obtained using both qualitative and semi quantitative methods to isolate quinolone resistant *E. coli* from dust, wipe and slurry samples from farm environments. For the qualitative method, an average of 1.91 g of sample was diluted 1:10 in Enterobacteriaceae Enrichment (EE) broth (Becton, Dickinson, Heidelberg, Germany) and incubated at 37 °C overnight. The enrichment was then spread on RAPID’*E. coli* agar (Biorad, Munich, Germany), supplemented with 8 μg/ml nalidixic acid, and incubated overnight at 37 °C. Using the semi-quantitative approach, an average of 1.97 g of sample, were diluted in a ratio of 1:10 in 0.85% saline solution and homogenized in a Stomacher sample blender (Seward Medical Ltd., London, UK). The homogenate was spread in dilution steps of 1:100 and 1:1000, respectively, on RAPID’*E. coli* agar plates supplemented with 8 μg/ml nalidixic acid. One *E. coli* isolate randomly selected from each positive sample was collected for further analysis. Isolates with questionable identity on RAPID’*E. coli* agar were confirmed by matrix-assisted laser desorption/ionization time-of- flight mass spectrometry (MALDI-TOF–MS, Bruker Daltronics, Bremen, Germany).

In total, the strains originated from dust (*n* = 48), wipes (*n* = 56), and slurry (*n* = 70), collected from 55 different farms (24 farrowing and rearing farms, 23 fattening farms and 8 mating and gestation farms) located in central and north-eastern Switzerland. All farms were part of a sow pool system. Of the 55 farms, 23 (41.8%) reported use of fluoroquinolones during the study period of 2016, including 16 (66.7%) of the farrowing and rearing, four (17.4%) of the fattening, and three (37.5%) of the mating and gestation farms.

### Microbiological methods

#### Antimicrobial susceptibility testing

The antimicrobial resistance profiles of the isolates were determined using the disk diffusion (Kirby Bauer) method according to the Clinical and Laboratory Standards Institute (CLSI) performance standards and breakpoints for human clinical isolates [17]. Mueller-Hinton agar culture medium (Becton Dickinson, Allschwil, Switzerland) was inoculated with a saline suspension of isolated colonies adjusted to 0.5 McFarland turbidity standard. Antibiotic disks (Becton Dickinson and company, Sparks, MD USA) were used, containing 30 μg nalidixic acid (NA), 5 μg ciprofloxacin (CIP), 10 μg ampicillin (AM), 20 μg/10 μg amoxicillin/clavulanic acid (AMC), 30 μg cefazolin (CZ), 30 μg cefotaxime (CTX), 30 μg cefepime (FEP), 23.75 μg /1.25 μg sulfamethoxazole/trimethoprim (SXT), 30 μg chloramphenicol (C), 15 μg azithromycin (AZM), 30 μg tetracycline (TE), 10 μg streptomycin (STR), 30 μg kanamycin (K), 10 μg gentamicin (GM), 300 μg nitrofurantoin (FM), and 200 μg fosfomycin (FOS). After 18 h of incubation at 35 °C ± 2 °C, results were interpreted as either sensitive (S), intermediate (IR), or resistant (R) according to the zone diameters around the disks using CLSI breakpoints [17]. Results were confirmed to be within the quality control ranges described by CLSI for *E. coli* ATCC25922 [17].

Isolates displaying resistance to three or more classes of antimicrobials (counting β-lactams as one class) were defined as multidrug-resistant (MDR).

### Molecular methods

#### Analysis of the quinolone resistance-determining region (QRDR) in *gyrA*

All strains were examined for mutations in the quinolone resistance-determining regions (QRDRs) of *gyrA*, using PCR amplification and sequencing primers as described previously [18]. Synthesis of primers and DNA custom sequencing was carried out by Microsynth (Balgach, Switzerland). Nucleotide sequences were analyzed with CLC Main Workbench 8.0.1 and aligned with the sequence *gyrA* reference strain *E. coli* K-12, substrain MG1655 (GenBank: U00096). For database searches the BLASTN program of the National Centre for Biotechnology Information (NCBI) (http://www.ncbi.nlm.nih.gov/blast/) was used.

#### Detection of plasmid mediated quinolone resistance genes (PMQR)

The plasmid-mediated fluoroquinolone resistance genes *aac(6′)-Ib-cr*, *qnrA*, *qnrB*, *qnrC*, *qnrD*, *qnrS*, and *qepA* were detected by PCR as described elsewhere [19–25]. Synthesis of primers and DNA custom sequencing was carried out by Microsynth (Balgach, Switzerland) and nucleotide sequences were analyzed with CLC Main Workbench 8.0.1. In addition, *qnrB* and *qnrS* genes were sequenced.

The presence of *qnrB* was confirmed by PCR as described by Abgottspon et al., using strain N05–2379 as a positive control [26]. Purified amplicons were custom sequenced (Microsynth, Balgach, CH) using the forward primer (qnrB_Seq_F) [26] . Sequences were analysed using the Basic Local Alignment Search Tool (BLAST) of the NCBI (https://blast.ncbi.nlm.nih.gov/Blast.cgi) (Bethesda, USA).

The presence of *qnrS* confirmed and sequenced as described by Zurfluh et al., using *E. coli* OW95E1 as a positive control and the forward primer (qnrS_orf_F) [27]. Sequences were analysed as described for *qnrB*.

#### Phylogenetic characterization and multilocus sequence typing

Phylogenetic classification of the *E. coli* isolates into one of the eight groups A, B1, B2, C, D, E, F (*E. coli* sensu stricto), or *Escherichia* clade I, was performed as described by Clermont et al. [28].

Sequence type (ST) determination of the *E. coli* isolates was carried out as described by Wirth et al. [29]. Sequences were imported into the *E. coli* multilocus sequence type (MLST) database (http://enterobase.warwick.ac.uk) to determine MLST types and clonal complexes (CC).

#### Serotyping of *E. coli* ST301

Strains belonging to CC165 and ST301 frequently belong to the unusual O80 serogroup [30]. To test this possibility, all isolates belonging to ST301 were serotyped. The O80 serogroup was identified by O80-specific PCR using primers and conditions described previously [30]. The H2 type was determined by PCR targeting the *flicH2* gene with primers described elsewhere [31]. The presence of the intestinal virulence genes *stx*, *eae* and of extra-intestinal virulence genes associated with plasmid pS88 [32] was evaluated as described previously [33].

## Results

### Antimicrobial resistance phenotypes

Antimicrobial susceptibility testing by the disc diffusion method showed that 81% (*n* = 141) of the strains were resistant and 19% (*n* = 33) were intermediately resistant to nalidixic acid (Table 1). Furthermore, 36.2% (*n* = 63) of the isolates were also resistant to ciprofloxacin (Table 1).

**Table 1:** *Escherichia coli* isolated from dust, wipes and slurry samples from pig farms

Isolate ID	Farm ID	Farm type	FQ usage	Source	NA	CIP	QRDR *gyrA*	PMQR gene	PG	ST	CC	Additional resistance
19	11	F&R	**+**	D	R	IR	S83L, D87N	–	B1	453	86	AM, SXT, TE, STR, GM
45	12	F&R	**+**	D	R	R	S83L, D87N	–	B1	162	469	AM, SXT, TE, STR, K
22	13	F&R	**+**	D	R	R	S83L, D87N	–	B1	1642	–	AM, SXT, C, TE, STR, K, GM
28	13	F&R	**+**	D	R	R	S83L, D87N	–	B1	1642	–	AM, SXT, C, TE, STR, K, GM
40	17	F&R	**+**	D	R	R	S83L, D87N	–	A	10	10	AM, SXT, C, TE, STR
44	17	F&R	**+**	D	R	R	S83L, D87N	–	B1	453	86	AM, SXT, TE, STR, K, GM
8	18	F&R	**+**	D	R	R	S83L, D87N	–	A	2197	–	AM, SXT, AZM, C, TE, STR, K, GM
9	18	F&R	**+**	D	R	R	S83L, D87N	–	A	2197	–	AM, CTX, SXT, AZM, C, TE, STR, K, GM
54	21	F&R	**+**	D	R	R	S83L, D87N	–	A	2197	–	AM, SXT, C, TE, STR, GM
55	21	F&R	**+**	D	R	S	S83L	–	B1	345	–	AM, SXT, STR, K
117	22	F&R	**+**	D	R	S	S83L	–	C	90	23	–
126	23	F&R	**+**	D	R	S	S83L	–	A	898	–	TE
127	23	F&R	**+**	D	R	S	S83L	–	A	898	–	–
129	23	F&R	**+**	D	R	S	S83L	–	A	898	–	–
62	29	F&R	**+**	D	R	R	S83L, D87N	*aac(6′)-Ib-cr*	C	6332	–	AM, AMC, CZ, CTX, FEP, SXT, AZM, TE, STR, GM
149	29	F&R	**+**	D	IR	S	–	*qnrS*	A	542	–	SXT, TE, STR
66	30	F&R	**+**	D	R	R	S83L, D87N	–	B1	453	86	AM, SXT, TE, STR, K, GM
160	30	F&R	**+**	D	IR	S	–	*qnrB*	A	new	–	AM, SXT, TE, STR
168	30	F&R	**+**	D	IR	S	–	*qnrB*	A	34	10	TE, STR
169	30	F&R	**+**	D	IR	S	–	*qnrB*	A	43	10	–
2	71	F&R	**+**	D	R	R	S83L, D87N	–	B1	297	–	AM, AMC
11	71	F&R	**+**	D	R	S	S83L	*qnrS*	A	2496	–	AM, AMC, STR
145	71	F&R	**+**	D	IR	IR	–	*qnrS*	A	301	165	SXT, TE, STR
107	73	F&R	**+**	D	R	S	S83L	–	A	10	10	AM, C, TE, STR
109	73	F&R	**+**	D	R	S	S83L	–	A	898	–	TE, STR
97	69	FF	**+**	D	R	R	S83L, D87N	–	B1	297	–	TE
173	69	FF	**+**	D	IR	S	–	*qnrB*	E	1607	–	–
113	1	M&G	**+**	D	R	S	S83L	*qnrS*	A	10	10	AM, SXT, STR
164	1	M&G	**+**	D	IR	S	–	*qnrS*	A	new	–	AM
13	6	M&G	**+**	D	R	S	S83L	–	B1	737	–	SXT, TE, STR
14	6	M&G	**+**	D	R	S	S83L	–	B1	737	–	SXT, TE, STR
41	17	F&R	**+**	W	R	R	S83L, D87N	–	B1	58	155	AM, SXT, TE, STR
125	22	F&R	**+**	W	R	S	S83L	–	A	4691	–	STR
56	23	F&R	**+**	W	R	S	S83L	–	A	1684	–	SXT, STR
128	23	F&R	**+**	W	R	S	S83L	–	C	410	23	TE, STR, K
59	26	F&R	**+**	W	R	S	S83L	–	E	302	–	AM, STR
139	26	F&R	**+**	W	R	S	S83L	–	E	302	–	AM, STR
65	29	F&R	**+**	W	R	S	–	*qnrB*	E	1607	–	SXT, AZM, STR
161	29	F&R	**+**	W	IR	S	–	*qnrB*	E	1607	–	–
67	30	F&R	**+**	W	R	S	S83L	–	A	746	–	SXT, TE, STR
141	30	F&R	**+**	W	R	S	S83L	–	E	302	–	–
143	30	F&R	**+**	W	IR	S	–	–	E	302	–	–
150	30	F&R	**+**	W	IR	S	–	*qnrB*	A	10	10	SXT, FM, STR
3	71	F&R	**+**	W	R	S	S83L	–	A	542	–	–
12	71	F&R	**+**	W	R	IR	S83L	qnrS	A	2496	–	AM, CZ, STR
137	71	F&R	**+**	W	R	S	D87G	–	A	3630	–	–
144	71	F&R	**+**	W	IR	IR	–	*qnrS*	E	new	–	AM, SXT, TE, STR
99	73	F&R	**+**	W	R	R	S83L, D87N	–	B1	453	86	AM, SXT, TE, STR
101	73	F&R	**+**	W	R	S	S83L	–	A	898	–	SXT, TE, STR
102	73	F&R	**+**	W	R	S	S83L	–	A	1684	–	SXT, TE, STR
106	73	F&R	**+**	W	R	S	–	*qnrS*	A	871	–	SXT, TE, STR
123	73	F&R	**+**	W	R	S	S83L	–	B1	3695	–	–
155	74	F&R	**+**	W	IR	S	–	*–*	C	23	23	TE, STR, K
119	38	FF	**+**	W	R	S	S83L	–	A	100	165	SXT, TE, STR
152	53	FF	**+**	W	IR	S	–	*qnrB* + *qnrS*	E	new	–	AM10, SXT, TE, STR
146	59	FF	**+**	W	IR	S	–	*qnrB* + *qnrS*	A	10	10	AM, SXT, TE, STR, K, GM
132	69	FF	**+**	W	R	S	S83L, D87N	–	B1	297	–	SXT, TE, STR
163	1	M&G	**+**	W	IR	S	–	*qnrB*	A	34	10	TE, STR
157	4	M&G	**+**	W	IR	S	–	*qnrB*	A	1602	–	STR
158	4	M&G	**+**	W	IR	S	–	*qnrB*	A	new	–	STR
115	6	M&G	**+**	W	R	S	S83L	–	D	362	–	AM, SXT, C, TE, STR, K
124	6	M&G	**+**	W	R	S	S83L	–	A	93	168	AM, SXT, FOS, STR
20	12	F&R	**+**	SL	R	IR	S83L, D87N	–	B1	297	–	TE
21	12	F&R	**+**	SL	R	IR	S83L, D87N	–	B1	297	–	TE
27	12	F&R	**+**	SL	R	IR	S83L, D87Y	–	A	10	10	–
23	13	F&R	**+**	SL	R	S	D87Y	–	A	34	10	STR
24	13	F&R	**+**	SL	R	R	S83L, D87N	–	B1	453	86	AM, SXT, TE, STR, K, GM
29	13	F&R	**+**	SL	R	S	D87Y	–	A	34	10	STR
30	13	F&R	**+**	SL	R	S	S83L	–	A	301	165	SXT, STR
31	13	F&R	**+**	SL	R	S	S83L	–	A	301	165	SXT, AZM, STR
25	16	F&R	**+**	SL	R	S	S83L	–	A	10	10	–
26	16	F&R	**+**	SL	R	R	S83L, D87N	–	B1	297	–	FOS, TE
42	16	F&R	**+**	SL	R	R	S83L, D87N	–	B1	297	–	TE
116	16	F&R	**+**	SL	R	S	S83L	–	A	10	10	–
165	16	F&R	**+**	SL	R	R	S83L, D87N	*–*	B1	297	–	TE
38	17	F&R	**+**	SL	R	R	S83L, D87N	–	B1	297	–	–
39	17	F&R	**+**	SL	R	R	S83L, D87N	–	B1	453	86	AM, SXT, TE, STR, GM
43	17	F&R	**+**	SL	R	R	S83L, D87N	–	B1	453	86	AM, SXT, TE, STR, K, GM
156	18	F&R	**+**	SL	IR	S	–	*qnrB*	A	77	206	AM, SXT, C, STR
166	21	F&R	**+**	SL	IR	S	–	*qnrB*	A	10	10	SXT, STR
118	26	F&R	**+**	SL	R	S	S83L	–	A	6593	165	STR
130	28	F&R	**+**	SL	R	S	S83L	–	A	93	168	–
136	28	F&R	**+**	SL	R	S	S83L	–	A	93	168	–
167	28	F&R	**+**	SL	IR	S	–	*qnrS*	A	48	10	AM, TE, STR
63	29	F&R	**+**	SL	R	R	S83L, D87N	–	B1	453	86	AM, SXT, TE, STR
64	29	F&R	**+**	SL	R	R	S83L, D87N	–	B1	453	86	AM, SXT, TE, STR
4	71	F&R	**+**	SL	R	R	S83L, D87N	–	A	3902	–	AM, SXT, TE, STR
5	71	F&R	**+**	SL	R	IR	S83L	*qnrS*	A	2496	–	AM, CTX, FOS, AZM, STR
6	71	F&R	**+**	SL	R	IR	S83L, D87N	–	B1	162	469	AM, SXT, TE, STR, K
7	71	F&R	**+**	SL	R	R	S83L, D87N	–	B1	162	469	AM, SXT, TE, STR, K
10	71	F&R	**+**	SL	R	IR	S83L	*qnrS*	A	2496	–	AM, STR
100	73	F&R	**+**	SL	R	S	S83L	–	A	10	10	AM, C, TE, STR
108	73	F&R	**+**	SL	R	S	S83L	–	A	10	10	AM, C, TE, STR
122	73	F&R	**+**	SL	R	S	S83L	–	A	10	10	AM, C, TE, STR
85	53	FF	**+**	SL	R	R	S83L, D87N	–	B1	162	469	STR
96	69	FF	**+**	SL	R	R	S83L, D87N	–	B1	297	–	TE
1	1	M&G	**+**	SL	R	S	S83L	–	B1	156	156	SXT, STR
15	6	M&G	**+**	SL	R	R	S83L	–	B1	737	–	SXT, TE, STR
114	6	M&G	**+**	SL	R	S	S83L	–	B1	737	–	SXT, TE, STR
135	6	M&G	**+**	SL	R	S	S83L	–	A	93	168	–
34	14	F&R	–	D	R	R	S83L, D87N	–	E	1011	–	AM
35	14	F&R	–	D	R	R	S83L, D87N	–	E	1011	–	AM
49	20	F&R	–	D	R	R	S83L, D87N	–	A	744	–	AM, SXT, C, TE, STR, K, GM
53	20	F&R	–	D	R	IR	S83L, D87N	–	A	2197	–	AM, SXT, C, TE, STR, GM
138	20	F&R	–	D	R	S	S83L	–	B1	58	155	AM, SXT, TE, STR
50	24	F&R	–	D	R	R	S83L, D87N	–	A	10	10	AM, SXT, C, TE, STR
57	25	F&R	–	D	R	R	S83L, D87N	–	A	744	–	AM, SXT, C, TE, STR, K
60	25	F&R	–	D	R	R	S83L, D87N	–	A	744	–	AM, SXT, C, TE, STR, K
105	70	F&R	–	D	R	S	S83L	–	B1	847	–	AM, SXT, STR, K
98	72	F&R	–	D	R	R	S83L, D87N	–	B1	297	–	AM, CTX
104	72	F&R	–	D	R	R	S83 L, D87N	–	B1	new	–	SXT, TE, GM
82	52	FF	–	D	R	R	S83L, D87N	–	B1	1431	–	SXT
87	54	FF	–	D	R	S	S83L	–	C	88	23	AM, SXT, TE, STR, K
153	57	FF	–	D	IR	S	–	*qnrB*	B1	99	–	TE, STR
133	61	FF	–	D	R	S	S83L	–	A	93	168	–
95	67	FF	–	D	R	S	S83L	–	B1	737	–	SXT, TE, STR
112	78	M&G	–	D	R	R	S83L, D87N	–	C	90	23	AM, SXT, TE, STR, GM
32	14	F&R	–	W	R	S	S83L	–	C	88	23	AM, SXT, STR, K
33	14	F&R	–	W	R	S	S83L	–	C	88	23	AM, SXT, STR, K
48	20	F&R	–	W	R	R	S83L, D87N	–	A	2197	–	AM, SXT, C, TE, STR, GM
51	20	F&R	–	W	R	R	S83L, D87N	–	B1	297	–	TE
52	20	F&R	–	W	R	R	S83L, D87N	–	B1	297	–	TE
46	24	F&R	–	W	R	R	S83L, D87N	–	A	10	10	AM, SXT, C, TE, STR
140	70	F&R	–	W	IR	S	–	–	F	117	–	–
120	76	F&R	–	W	R	S	S83L	–	C	23	23	–
61	35	FF	–	W	R	S	S83L	–	B1	847	–	SXT, TE, STR, GM
70	35	FF	–	W	R	S	S83L	–	B1	new	–	SXT, TE, STR
162	35	FF	–	W	IR	S	–	*qnrS*	A	227	10	TE, STR, K
76	47	FF	–	W	R	S	S83L	–	A	898	–	TE, STR
74	50	FF	–	W	R	R	S83L, D87N	–	B1	297	–	TE
78	50	FF	–	W	R	R	S83L, D87N	–	A	2509	–	AZM, TE, STR
80	50	FF	–	W	R	R	S83L, D87N	–	B1	297	–	TE
81	52	FF	–	W	R	R	S83L, D87N	–	B1	297	–	TE
86	54	FF	–	W	R	S	S83L	–	C	88	23	AM, SXT, TE, STR, K
89	58	FF	–	W	R	S	S83L	–	B1	58	155	SXT, TE, STR
91	63	FF	–	W	R	S	S83L	–	C	88	23	AM, SXT, STR, K
90	65	FF	–	W	R	R	S83L, D87N	–	B1	297	–	TE
94	66	FF	–	W	R	R	S83L, D87N	–	B1	297	–	TE
172	68	FF	–	W	IR	S	–	*qnrB*	A	993	–	TE, STR
147	7	M&G	–	W	IR	IR	–	*qnrB* + *qnrS*	A	10	10	AM, SXT, TE, STR, GM
148	7	M&G	–	W	IR	S	–	*qnrB* + *qnrS*	A	10	10	AM10, SXT, TE, STR, GM
174	78	M&G	–	W	IR	S	–	*qnrB*	B1	3322	86	AM, STR, GM
36	14	F&R	–	SL	R	S	S83L	–	F	117	–	AM, SXT, C, TE, STR
37	14	F&R	–	SL	R	R	S83L, D87N	–	A	2197	–	AM, SXT, C, TE, STR, GM
47	24	F&R	–	SL	R	R	S83L, D87N	–	A	10	10	AM, SXT, C, TE, STR
58	25	F&R	–	SL	R	R	S83L, D87N	–	A	744	–	AM, SXT, C, TE, STR, K
68	33	F&R	–	SL	R	R	S83L, D87N	–	A	2509	–	AM, TE, STR
69	33	F&R	–	SL	R	R	S83L, D87N	–	A	2509	–	AM, TE, STR
103	72	F&R	–	SL	R	R	S83L, D87N	–	A	10	10	AM, AMC, SXT, C, TE, STR, K
151	35	FF	–	SL	IR	S	–	*qnrB*	B1	1665	–	K
71	37	FF	–	SL	R	R	S83L, D87N	–	A	2509	–	TE, STR
121	42	FF	–	SL	R	S	S83L	–	B1	1157	–	AM, SXT, TE, STR
75	45	FF	–	SL	R	S	S83L	–	C	88	23	AM, SXT, STR
73	47	FF	–	SL	R	S	S83L	–	A	898	–	TE, STR
77	47	FF	–	SL	R	S	S83L	–	A	898	–	TE, STR
79	49	FF	–	SL	R	R	S83L, D87N	–	B1	297	–	SXT, TE, STR, K, GM
72	50	FF	–	SL	R	R	S83L, D87N	–	A	2509	–	TE, STR
170	50	FF	–	SL	IR	S	–	*qnrB*	B1	99	–	TE, STR
83	52	FF	–	SL	R	R	S83L, D87N	–	B1	297	–	TE
154	57	FF	–	SL	IR	S	–	*qnrS*	A	542	–	TE, STR, K
84	58	FF	–	SL	R	S	S83L	–	C	88	23	AM, SXT, STR, K
88	58	FF	–	SL	R	S	S83L	–	C	88	23	AM, SXT, STR, K
171	63	FF	–	SL	IR	S	–	*qnrB*	E	524	32	–
93	64	FF	–	SL	R	R	S83L, D87N	–	B1	453	86	AM, SXT, TE, STR
92	65	FF	–	SL	R	S	S83L	–	C	88	23	AM, SXT, STR, K
131	68	FF	–	SL	R	S	S83L	–	A	93	168	–
111	77	FF	–	SL	R	R	S83L, D87N	–	B1	1196	–	AM, SXT, C, TE, STR
134	77	FF	–	SL	R	S	S83L	–	A	10	10	AM, C, STR
159	8	M&G	–	SL	IR	S	–	*qnrB*	A	4429	–	STR
16	9	M&G	–	SL	R	R	S83L, D87N	–	A	10	10	AM, SXT, C, TE, STR
17	9	M&G	–	SL	R	R	S83L, D87N	–	C	410	23	TE
18	10	M&G	–	SL	R	S	S83L	–	A	6593	165	STR
142	10	M&G	–	SL	IR	S	–	*qnrB*	A	48	10	STR
110	78	M&G	–	SL	R	R	S83L, D87N	–	C	90	23	AM, SXT, TE, STR, GM

*AM* ampicillin, *AMC* amoxicillin with clavulanic acid, *AZM* azithromycin, *C* chloramphenicol, *CC* clonal complex, *CIP* ciprofloxacin, *CTX* cefotaxime, *CZ* cefazolin, *D* dust sample, *F&R* farrowing and rearing farm, *FEP* cefepime, *FF* fattening farm, *FM* nitrofurantoin, *FOS* fosfomycin, *GM* gentamicin, *IR* intermediately resistant, *K* kanamycin, *M&G* mating and gestation farm, *NA* nalidixic acid, *new* new combination of alleles (detailed in Additional file 1: Table A1); *PG* phylogenetic group, *PMQR* plasmid-mediated quinolone resistance, *QRDR* quinolone resistance-determining region, *R* resistant, *S* susceptible, *STR* streptomycin, *SL* slurry sample, *SXT* sulfamethoxazole/trimethoprim, *ST* sequence type, *TE* tetracycline, *W* wipe sample; +, use of fluoroquinolone during study period 2016; −, no use of fluoroquinolone during study period 2016; −–, absence of point mutation, gene, CC, or additional resistance

Additional antimicrobial resistance was most frequently observed for streptomycin (72.4% /*n* = 126), tetracycline (60.9% /*n* = 106), sulfamethoxazole/trimethoprim (50% /*n* = 87), ampicillin (46.6% /*n* = 81), kanamycin (19.5% /*n* = 34), chloramphenicol (15.5% /*n* = 27), and gentamicin (14.4% /*n* = 25), respectively (Table 1). Resistance to all other tested antibiotics was detected for at least one isolate, except to nitrofurantoin (Table 1).

Of the 174 isolates analysed in this study, 68.4% (*n* = 119) were resistant to three or more classes of antibiotics and therefore categorised as MDR. The most frequent MDR combinations detected were SXT-TE-STR (*n* = 15), AM-SXT-TE-STR (*n* = 10) and AM-SXT-STR-K (*n* = 8) (Table 1). *E. coli* strains resistant to four and five antibiotics were the most prevalent (21.3 and 19.0%, respectively).

### Molecular properties

Of 141 isolates with a nalidixic acid resistant phenotype, 98.6% (*n* = 139) possessed at least one nucleotide mutation in the QRDR of *gyrA*. Thereof, 49.6% (*n* = 70) showed single amino acid substitution at codon Ser83, namely Ser83 to Leu (*n* = 67), or Asp87 to Tyr (*n* = 2), or Asp87 to Gly (n = 1). Further, 48.9% (*n* = 69) possessed double substitutions at Ser83 to Leu and Asp87 to Asn (*n* = 68) or Tyr (n = 1). Two isolates (isolates no. 65 and 106, respectively) tested negative for mutations in the QRDR of *gyrA* (Table 1).

A total of 38 strains possessed one or more PMQR genes, representing 21.8% of the 174 analysed strains (Table 1). Among the 19.5% (*n* = 34) of the isolates with one PMQR gene, twenty (11.5%) possessed *qnrB,* thirteen (7.5%) *qnrS* and one isolate (0.6%) possessed *aac(6′)-Ib,* respectively (Table 1). Four isolates (2.3%) possessed a combination of *qnrB* and *qnrS* genes. No isolates tested positive for *qnrA*, *qnrC*, *qnrD* or *qepA*. The occurrence of PMQR positive isolates was remarkably higher in strains exhibiting intermediate resistance to nalidixic acid (90.9% /*n* = 30), than in nalidixic acid resistant strains (5.7% /*n* = 8). Moreover, all *qnrB*/*qnrS* combinations were detected in intermediately resistant isolates (Table 1). Isolates possessing PMQR were found in 11 (22.9%) of the dust samples 16 (28.6%) of the wipe samples. and 11 (15.7%) of the slurry samples (Table 1).

Of the 23 farms with reported use of fluoroquinolones, 12 (52.2%) yielded environmental *E. coli* containing PMQR genes. Thereof, the majority (7 farms/58.3%) were farrowing and rearing farms, three (25%) were fattening farms and two (16.7%) were mating and gestation farms (Table 1).

By contrast, of the 32 farms without a history of fluoroquinolone use during the study period, nine (28.1%) tested positive for *E. coli* harbouring PMQR genes. Thereof, five (55.6%) were fattening farms, four (44.4%) were mating and gestation farms, and none (0%) were farrowing and rearing farms (Table 1).

### Phylogenetic grouping

The majority of the isolates were assigned to phylogenetic groups A (48.3%/*n* = 84) and group B1 (33.3% /*n* = 58). The remaining strains were classified into group C (9.8% /*n* = 17), E (6.9%/*n* = 12), F (1.1%/*n* = 2) and D (0.6%/n = 1), respectively (Table 1). None of the isolates belonged to phylogenetic group B2.

### MLST

Overall, a total of 50 STs were found. The most common sequence types were ST10 (*n* = 20), ST297 (n = 20), ST453 (*n* = 10), ST88 (*n* = 9), ST898 (n = 8), ST93 (*n* = 6), ST2197 (n = 6), ST737 (n = 5), and ST2509 (n = 5) (Table 1). Seven isolates could not be assigned to any known ST, because the allele combinations were new (Table 1). The allele combinations are listed in Additional file 1: Table A1.

### Characteristics of *E. coli* ST301 isolates

Three isolates belonged to ST301 (Table 1). Thereof, two (isolates 30 and 31, respectively) belonged to serotype O80:H2 and possessed the *eae*-ξ variant. None of the isolates harboured *stx* or any genes related to pS88. Both isolates were therefore classified as enteropathogenic *E.coli* (EPEC).

## Discussion

### Resistance profiles

In the present study, we determined the prevalence of point mutations within the QRDR of *gyrA* and the presence of PMQR genes among 174 *E. coli* isolated from pig farm environments. All isolates were non-susceptible to quinolones and, using the disk diffusion method, were classified as intermediate or as resistant to nalidixic acid.

All resistant isolates except two, possessed at least one mutation in *gyrA*. Notably, both these isolates possessed a PMQR gene, (isolate 65 possessed *qnrB*, and isolate 106 *qnrS*, respectively). Since *qnr* genes alone are insufficient to confer resistance [34–36], further mechanisms are likely associated with the resistance phenotype of these strains, such as mutations in the QRDR regions of the *gyrB*, *parC* genes, or increased efflux pump activity (both not evaluated in this study).

Of the 70 nalidixic acid resistant strains possessing one mutation in *gyrA*, only one (0.7%) was resistant to ciprofloxacin. By contrast, of the isolates with double mutations in *gyrA*, the majority (89.9%) were also resistant to ciprofloxacin. These observations correlate with previous studies that link the number of quinolone resistance-associated mutations and the resistance phenotype of an isolate [37, 38].

In this study, only a minority of the resistant strains carried PMQR genes, and, except for *aac(6′)-Ib-cr*, none of the PMQR genes were associated with ciprofloxacin resistance. Isolates with *qnrS* also possessed a Ser83 to Leu substitution in the *gyrA* QRDR and showed decreased susceptibility to ciprofloxacin. Two further resistant isolates harbouring *qnrS* or *qnrB* lacked mutations in the *gyrA* QRDR and were susceptible to ciprofloxacin. In short, these data correlate with results from previous studies on the coexistence of different resistance mechanisms in one isolate [37–39].

Notably, the vast majority (90.9%) of strains with intermediate resistance to nalidixic acid were associated with the presence of *qnrB*, *qnrS* or a combination thereof. The three remaining intermediately resistant isolates lacking both *gyrA* mutations and *aac(6′)-Ib-cr*, *qnrA*, *qnrB*, *qnrC*, *qnrD*, *qnrS*, or *qepA* genes, are likely to possess other resistance-mechanisms that we did not screen for in the present study, e.g., point mutations within the QRDR of *gyrB* or in the topoisomerase genes *parC* or *parE* [11]. Four isolates possessed a combination of *qnrB* and *qnrS* genes but remained intermediately resistant to nalidixic acid. These results are in agreement with the findings of other studies [40, 41], which demonstrate that the presence of two different *qnr* genes in the same strain has no additive effect on resistance levels. Further, our data correlate with previously mentioned studies concerning the coexistence of different resistance mechanisms in one isolate [37–39].

The predominance of PMQR among isolates that lack *gyrA* mutations is noteworthy, since these genes are known to facilitate the selection of resistant mutants. Data from other studies [42, 43] suggest that, depending on which mutations are already present in a strain, the acquisition of further fluoroquinolone resistance genes could increase the strain’s fitness.

In this study, the most frequent PMQR genes were *qnrB* and *qnrS*, respectively. Correlating with our data, it has been reported previously that *qnrB* is the most frequent PMQR gene, followed by *qnrS*. [11, 36, 44, 45]. The presence of *qnr* genes in environmental *E. coli* indicates that selection could occur without exposure to inhibitory concentrations of fluoroquinolones. It has been demonstrated previously for environmental *Klebsiella pneumoniae* isolated from wastewater that *qnr* genes confer a selective advantage in the presence of residual subinhibitory fluoroquinolone concentrations present in wastewater [46] Accordingly, pig farms with a history of (in-feed) application of fluoroquinolones may represent environments containing residual concentrations of antibiotics which propagate PMQR genes. Our data suggest that this may hold true in particular for farrowing and rearing farms, where such genes were detected exclusively on farms with a history of use of fluoroquinolones. Environmental pollution with residual fluoroquinolones is facilitated by their poor degradability and strong potential for binding to sediments [47]. In the absence of solar radiation some fluoroquinolones (enrofloxacin) remain stable for at least 120 days [48]. In their long-term experimental study Xu and colleagues [49] stated that the application of swine manure lead to an increase of fluoroquinolone resistances in soil, including PMQR genes, which persisted at least five months. On farms without prior treatment of animals, strains harbouring quinolone resistance genes may be introduced during transfer of pigs from other locations, comparable to inter-farm transmissions of extended-spectrum ß-lactamase (ESBL) producing *E. coli* in pigs [50]. Our data suggest that this may especially be the case for mating and gestation farms and for fattening farms, where, in contrast to farrowing and rearing farms, environmental contamination with *E. coli* harbouring PMQR genes was detected irrespective of the history of fluoroquinolone treatment. These findings suggest that animal movement to and from farrowing and rearing farms with recent histories of treatment may promote the risk of transmission of resistant bacteria and of fluoroquinolone resistance genes among farms within sow pool systems.

Apart from direct transmission of resistant *E. coli*, PMQR genes can be transferred horizontally to other bacteria in the pig farm environment. Exposure to quinolones of bacteria containing *qnr* genes may increase their capacity to acquire point mutations in the gyrase and/or topoisomerase IV genes [46]. PMQR genes are often harboured on plasmids containing other resistance genes, e.g., ß-lactamases [51], thus, the use of non-fluoroquinolone antimicrobials enables their co-selection [52].

Overall, the resistance profiles of the isolates described in this study are in agreement with previous studies that have demonstrated that some of the most common antibiotic resistances (other than to quinolones) in *E. coli* in the pig environment are to tetracycline, ampicillin, streptomycin and sulfamethoxazole [53, 54], and that *E. coli* strains from the pig environment often are resistant to four or five antibiotics simultaneously [55].

### Phylogenetic grouping, MLST and serotyping of ST301

The majority of the isolates belonged to phylogenetic groups A or B1. The predominance of ST10 among the *E. coli* belonging to group A reflect previous observations regarding isolates from pigs, chicken faeces, as well as retail chicken and pork meat [56, 57]. Furthermore, Araùjo et al. [58] observed that *E. coli* ST10 and ST297 were the predominant sequence types among MDR isolates isolated from irrigation water and vegetables in household farms, highlighting the wide dissemination of this sequence type and its association with MDR. Notably, *E. coli* ST10 (CC10), as well as *E. coli* CC23 also identified in this study, both including ciprofloxacin resistant and MDR strains, are also associated with urinary tract infection and sepsis in humans [59].

In addition, several other *E. coli* belonging to phylogenetic group A identified in this study have been associated with disease in food producing animals, such as avian pathogenic *E. coli* ST93 [60], *E. coli* ST744 isolated from diseased calves [61], and strains belonging to CC165 from food producing animals [62]. Notably, among the latter, we detected two EPEC O80:H2 isolates, which is considered an emerging pathogen among calves in Belgium [63]. This serotype has emerged among humans as a highly virulent extra-intestinal pathogenic Shiga-toxin producing *E. coli* (STEC) in France and Switzerland since 2015 [30, 33]. As opposed to the STEC O80:H2 found in humans, the isolates from this study lacked pS88-associated extra-intestinal virulence genes. Therefore, the relationship between EPEC O80:H2 isolated from the farm environment and human STEC isolates needs to be assessed, e.g. by whole genome sequencing, and the prevalence of EPEC O80:H2 in pigs should be established.

*E. coli* strains belonging to the phylogenetic group B1 are for the most part commensal, with the ability to persist in the environment [64]. Most ST of this phylogroup, such as ST58, ST162, or ST453 have been frequently detected among healthy livestock [65]. However, *E. coli* ST297, which was one of most frequently observed ST in this study, has been associated with disease in both poultry and humans [66]. Likewise, *E. coli* ST453 is known to cause extraintestinal disease in humans (urinary tract infections) and metritis in cattle [67].

A minority of strains were assigned to the extraintestinal virulent phylogroups D and F. Among these, we detected two MDR strains belonging to ST117 which is a well-recognized avian pathogenic *E*. *coli* with zoonotic potential [68].

## Conclusions

Quinolone non-susceptible *E. coli* are widespread in the environment of Swiss pig farms. In particular, isolates showing intermediate resistance to nalidixic acid frequently possess transmissible PMQR genes. This is worrisome, since the presence of *qnr* genes may increase the ability of bacteria to acquire point mutations in the gyrase and topoisomerase IV genes, resulting in high level resistance to (fluoro)quinolones. Furthermore, plasmids harbouring *qnr* genes may contribute to the horizontal spread of antibiotic resistance in livestock and in the environment. In pig farms which are part of sow pool systems, inter-farm measures that aim to reduce the risk of spreading resistant bacteria and resistance genes from one stage of production to the next need to be assessed and promoted. Our data further show that farm environments contain commensal MDR *E. coli* as well as *E. coli* with zoonotic potential. In particular, we demonstrate for the first time the presence of EPEC O80:H2 in an environmental sample from a pig farm.

In order to preserve the usefulness of fluoroquinolones and to protect animal and human health, surveillance of antimicrobial resistance is warranted. Measures for prudent use of (fluoro)quinolones as provided by the European Union’s guidelines for use of antimicrobials in veterinary medicine are of utmost importance [69].

## Additional file

Additional file 1: **Table A1.** Results of the MLST analysis for *E. coli* strains with new STs. (DOCX 26 kb)

## References

[1] Fairbrother JM, Nadeau E, Gyle CL (2005). *Escherichia coli* in postweaning diarrhea in pigs: an update on bacterial types, pathogenesis, and prevention strategies. Anim Health Res Rev.

[2] Imberechts H, De Greve H, Lintermans P. The pathogenesis of edema disease in pigs. A review. Vet Microbiol. 1992;32(2–3):221–33. 10.1016/0378-1135(92)90080-d 1626371

[3] Society of Swiss Veterinaries (GST) and the Federal Food Safety and Veterinary Office (BLV). *Umsichtiger Einsatz von Antibiotika: Therapieleitfaden für Tierärztinnen und Tierärzte*. (2018). Available at: https://www.blv.admin.ch/blv/de/home/tiere/tierarzneimittel/antibiotika/nationale-strategie-antibiotikaresistenzen%2D%2Dstar%2D%2D/sachgemaesser-antibiotikaeinsatz.html [Accessed December 14, 2018].

[4] World Health Organization (WHO). Critically important antimicrobials for human medicine, 5th revision 2016; 2017. Available at https://apps.who.int/iris/bitstream/handle/10665/255027/9789241512220eng.pdf;jsessionid=DD55AE12145788EE9B13F8D9676A4587?sequence=1.

[5] European Commission on Health and Food Safety: Measures to Tackle Antimicrobial Resistance Through the Prudent Use of Antimicrobials in Animals (2018). Available at https://publications.europa.eu/en/publication-detail/-/publication/aa676ddd-2d87-11e8-b5fe-01aa75ed71a1. Accessed 9 Jan 2019.

[6] Hamscher G, Pawelzick HT, Sczesny S, Nau H, Hartung J (2003). Antibiotics in dust originating from a pig-fattening farm: a new source of health hazard for farmers?. Environ Health Perspect.

[7] Jensen VF, Jakobsen L, Emborg HD, Seyfarth AM, Hammerum AM. Correlation between apramycin and gentamicin use in pigs and an increasing reservoir of gentamicin-resistant *Escherichia coli*. J Antimicrob Chemother. 2006;58(1):101–7. 10.1093/jac/dkl201 16709594

[8] Liu JH, Deng YT, Zeng ZL, Gao JH, Chen L, Arakawa Y, Chen ZL. Coprevalence of plasmid-mediated quinolone resistance determinants QepA, Qnr, and AAC(6′)-Ib-cr among 16S rRNA methylase RmtB-producing *Escherichia coli* isolates from pigs. Antimicrob Agents Chemother. 2008;52(8):2992–3. 10.1128/AAC.01686-07 PMC2493129 18490500

[9] Xia LN, Li L, Wu CM, Liu YQ, Tao XQ, Dai L, Qi YH, Lu LM, Shen JZ (2010). A survey of plasmid-mediated fluoroquinolone resistance genes from *Escherichia coli* isolates and their dissemination in Shandong. China Foodborne Pathog Dis.

[10] Cano ME, Rodríguez-Martínez JM, Agüero J, Pascual A, Calvo J, García-Lobo JM, Velasco C, Francia MV, Martínez-Martínez L (2009). Detection of plasmid-mediated quinolone resistance genes in clinical isolates of *Enterobacter* spp. in Spain. J Clin Microbiol.

[11] Strahilevitz J, Jacoby GA, Hooper DC, Robicsek A (2009). Plasmid-mediated quinolone resistance: a multifaceted threat. Clin Microbiol Rev.

[12] Chee-Sanford JC, Machkie RI, Koike S, Krapac IG, Lin Y, Yannarell AC, Maxwell S, Aminov RI (2008). Fate and transport of antibiotic residues and antibiotic resistance genes following land application of manure waste. J Eviron Qual.

[13] Heuer H, Schmitt H, Smalla K (2011). Antibiotic resistance gene spread due to manure application on agricultural fields. Curr Opin Microb.

[14] Jechalke S, Focks A, Rosendahl I, Groeneweg J, Siemens J, Heuer H, Smalla K (2014). Structural and functional response of the soil bacterial community to application of manure from difloxacin-treated pigs. FEMS Microb Eco.

[15] Sukul P, Lamshöft M, Kusari S, Zühlke S, Spiteller M (2009). Metabolism and excretion kinetics of 14C-labeled and non-labeled difloxacin in pigs after oral administration, and antimicrobial activity of manure containing difloxacin and its metabolites. Environ Res.

[16] Kruse H, Sorum H. Transfer of multiple drug resistance plasmids between bacteria of diverse origins in natural microenvironments. Appl Environ Microbiol. 1994:4015–21. 10.1128/aem.60.11.4015-4021.1994 PMC201930 11865872

[17] Clinical and Laboratory Standards Institute (CLSI). M100 – Performance standards for antimicrobial susceptibility testing (2016). CLSI supplement M100S, Wayne, PA 19087 USA.

[18] Rodriguez-Martinez JM, Velasco C, Pascual Á, Garcia I, Martinez-Martinez L (2006). Correlation of quinolone resistance levels and differences in basal and quinolone-induced expression from three *qnrA*-containing plasmids. Clin Microbiol Infect.

[19] Park CH, Robicsek A, Jacoby GA, Sahm D, Hooper DC. Prevalence in the United States of *aac(6′)-Ib-cr* encoding a ciprofloxacin-modifying enzyme. Antimicrob Agents Chemother. 2006:3953–5. 10.1128/AAC.00915-06 PMC1635235 16954321

[20] Karczmarczyk M (2010). Martins Marta, McCusker M, Mattar S, Amaral L, Leonard N, Aarestrup FM, fanning S. Characterization of antimicrobial resistance in *Salmonella enterica* food and animal isolates from Colombia: identification of a *qnrB19*-mediated quinolone resistance marker in two novel serovars. FEMS Microbiol Lett.

[21] Robicsek A, Strahilevitz J, Sahm DF, Jacoby GA, Hooper DC. *qnr *prevalence in ceftazidime-resistant *Enterobacteriaceae* isolates from the United States. Antimicrob Agents Chemother. 2006:2872–4. 10.1128/AAC.01647-05 PMC1538681 16870791

[22] Kim HB, Park CH, Kim CJ, Kim EC, Jacoby GA, Hooper DC. Prevalence of plasmid-mediated quinolone resistance determinants over a 9-year period. Antimicrob Agents Chemother. 2009:639–45. 10.1128/AAC.01051-08 PMC2630623 19064896

[23] Wang M, Guo Q, Xu X, Wang X, Ye X, Wu S, Hooper DC, Wang M (2009). New plasmid-mediated quinolone resistance gene, *qnrC*, found in a clinical isolate of *Proteus mirabilis*. Antimicrob Agents Chemother.

[24] Cavaco LM, Hasman H, Xia S, Aarestrup FM (2009). *qnrD*, a novel gene conferring transferable quinolone resistance in *Salmonella enterica* serovar Kentucky and Bovismorbificans strains of human origin. Antimicrob Agents Chemother.

[25] Cattoir V, Poirel L, Rotimi V, Soussy CJ, Nordmann P (2007). Multiplex PCR for detection of plasmid-mediated quinolone resistance qnr genes in ESBL-producing enterobacterial isolates. J Antimicrob Chemother.

[26] Abgottspon H, Zurfluh K, Nüesch-Inderbinen M, Hächler H, Stephan R. Quinolone resistance mechanisms in *Salmonella enterica* serovars Hadar, Kentucky, Virchow, Schwarzengrund, and 4,5,12:i:-, isolated from humans in Switzerland, and identification of a novel *qnrD* variant, *qnrD2*, in *S*. Hadar. Antimicrob Agents Chemother. 2014;58:3560–3. 10.1128/AAC.02404-14 PMC4068464 24733466

[27] Zurfluh K, Abgottspon H, Hächler H, Nüesch-Inderbinen M, Stephan R (2014). Quinolone resistance mechanisms among extended-spectrum beta-lactamase (ESBL) producing *Escherichia coli* isolated from rivers and lakes in Switzerland. PLoS One.

[28] Clermont O, Christenson JK, Denamur E, Gordon DM (2013). The Clermont *Escherichia coli* phylo-typing method revisited: improvement of specificity and detection of new phylo-groups. Environ Microbiol Rep.

[29] Wirth T, Falush D, Lan R, Colles F, Mensa P, Wieler LH, Karch H, Reeves PR, Maiden MCJ, Ochman H, Achtman M (2006). Sex and virulence in *Escherichia coli*: an evolutionary perspective. Mol Microbiol.

[30] Soysal N, Mariani-Kurkdjian P, Smail Y, Liguori S, Gouali M, Loukiadis E, Fach P, Bruyand M, Blanco J, Bidet P, Bonacorsi S (2016). Enterohemorrhagic *Escherichia coli* hybrid pathotype O80:H2 as a new therapeutic challenge. Emerg Infect Dis.

[31] Alonso CA, Mora A, Díaz D, Blanco M, González-Barrio D, Ruiz-Fons F, Simón C, Blanco J, Torres C (2017). Occurrence and characterization of *stx* and/or *eae*-positive *Escherichia coli* isolated from wildlife, including a typical EPEC strain from a wild boar. Vet Microbiol.

[32] Peigne C, Bidet P, Mahjoub-Messai F, Plainvert C, Barbe V, Médigue C, Frapy E, Nassif X, Denamur E, Bingen E (2009). The plasmid of *Escherichia coli* strain S88 (O45: K1: H7) that causes neonatal meningitis is closely related to avian pathogenic *E. coli* plasmids and is associated with high-level bacteremia in a neonatal rat meningitis model. Infect Immun.

[33] Nüesch-Inderbinen M, Cernela N, Wüthrich D, Egli A, Stephan R (2018). Genetic characterization of Shiga toxin producing *Escherichia coli* belonging to the emerging hybrid pathotype O80:H2 isolated from humans 2010-2017 in Switzerland. Int J Med Microbiol.

[34] Machuca J, Briales A, Díaz-de-Alba P, Martínez-Martínez L, Rodríguez-Martínez JM, Pascual Á (2016). Comparison of clinical categories for *Escherichia coli* harbouring specific *qnr* and chromosomal-mediated fluoroquinolone resistance determinants according to CLSI and EUCAST. Enferm Infecc Microbiol Clin.

[35] Rodríguez-Martínez JM, Briales A, Velasco C, Diaz de Alba P, Martínez-Martínez L, Pascual Á (2011). Discrepancies in fluoroquinolone clinical categories between the European committee on antimicrobial susceptibility testing (EUCAST) and CLSI for *Escherichia coli* harbouring *qnr* genes and mutations in *gyrA* and *parC*. J Antimicrob Chemother.

[36] Rodríguez-Martínez JM, Cano ME, Velasco C, Martínez-Martínez L, Pascual Á (2011). Plasmid-mediated quinolone resistance: an update. J Infect Chemother.

[37] Heisig P, Tschorny R (1994). Characterization of fluoroquinolone-resistant mutants of *Escherichia coli* selected *in vitro*. Antimicrob Agents Chemother.

[38] Lindgren PK, Karlsson A, Hughes D (2003). Mutation rate and evoultion of fluoroquinolone resistance in *Escherichia coli* isolates from patients with urinary tract infections. Antimicrob Agents Chemother.

[39] Jacoby GA (2005). Mechanisms of resistance to quinolones. Clin Infect Dis.

[40] Flach CF, Boulund F, Kristiansson E, Larsson DGJ (2013). Functional verification of computationally predicted *qnr* genes. Ann Clin Microb Antimicrob.

[41] Marcusson LL, Frimodt-Møller N, Hughes D. Interplay in the Selection of Fluoroquinolone Resistance and Bacterial Fitness. PLoS Pathog 2009;5(8):e1000541. 10.1371/journal.ppat.1000541. PMC2714960 19662169

[42] Marcusson LL, Frimodt-Møller N, Hughes D. Interplay in the Selection of fluoroquinolone-resistance and bacterial fitness. PLoS Pathog. 2009;5(8):e1000541. 10.1371/journal.ppat.1000541. PMC2714960 19662169

[43] Andersson DI, Hughes D (2010). Antibiotic resistance and its cost: is it possible to reverse resistance?. Nat Rev Microbiol.

[44] Poirel L, Cattoir V, Nordman P (2012). Plasmid-mediated quinolone resistance; interaction between human, animal, and environmental ecologies. Front Microbiol.

[45] Cattoir V, Nordmann P (2009). Plasmid-mediated quinolone resistance in gram-negative bacteral species: an update. Curr Medi Chem.

[46] Kaplan E, Marano RBM, Jurkevitch E, Cytryn E. Enhanced bacterial fitness under residual fluoroquinolone concentrations is associated with increased gene expression in wastewater-derived *qnr* plasmid-harboring strains. Front Microbiol. 2018;91176. 10.3389/fmicb.2018.01176. PMC6003256 29937755

[47] Marengo JR, Kok RA, O’Brien K, Velagaleti RR, Stamm JM (1997). Aerobic biodegradation of (14C)-sarafloxacin hydrochloride in soil. Environm Tox Chem.

[48] Wu YB, Wang ZS, Liao XD (2005). Study on the excretion of enrofloxacin in chicken and its degradation in chicken feces. Acta Vet Zoot Sin.

[49] Xu Y (2015). Y W, ma Q, Zhou H. Occurrence of (fluoro) quinolones and (fluoro) quinolone resistance in soil receiving swine manure for 11 years. Sci Total Environ.

[50] Schmithausen R, Schulze-Geisthoevel S, Heinemann C, Bierbaum G, Exner M, Petersen B, Steinhoff-Wagner J (2018). Reservoirs and transmission pathways of resistant indicator bacteria in the biotope pig stable and along the food chain: a review from a one health perspective. Sustainability..

[51] Dolejska M, Villa L, Minoia M, Guardabassi L, Carattoli A. Complete sequences of IncHI1 plasmids carrying *bla*_CTX-M-1_ and *qnrS1* in equine *Escherichia coli *provide new insights into plasmid evolution. J Antimicrob Chemother. 2014;69:2388–93. 10.1093/jac/dku172 24862095

[52] Ingram PR, Roger BA, Sidjabat HE, Gibson JS, Inglis TJJ. Co-selection may explain high rates of ciprofloxacin non-susceptible* Escherichia coli* from retail poultry reared without prior fluoroquinolone exposure. J Med Microbiol. 2013;62:1743–6. 10.1099/jmm.0.062729-0 24136884

[53] Lay KK, Koowattananukul C, Chansong N, Chuanchuen R (2012). Antimicrobial resistance, virulence, and phylogenetic characteristics of *Escherichia coli* isolates from clinically healthy swine. Foodb Pathog Dis.

[54] Rosager WN, Peter NJ, Erik Lind JS, Svend H, Matthew D, Steen PK (2017). Comparison of antimicrobial resistance in *E. coli* isolated from rectal and floor samples in pens with diarrhoeic nursery pigs in Denmark. Prev Vet Med.

[55] de la Torre E, Colello R, Fernández D, Etcherverría A, Di Conza J, Gutkind GO, Tapia MO, Dieguez SN, Soraci AL, Padola NL (2015). Multidrug resistance in *Escherichia coli* carrying integrons isolated from a pig farm with moderate antbiotic use. J Gen Appl Microbiol.

[56] Herrero-Fresno A, Larsen I, Olsen JE (2015). Genetic relatedness of commensal *Escherichia coli* from nursery pigs in intensive pig production in Denmark and molecular characterization of genetically different strains. J Appl Microbiol.

[57] Bergeron CR, Prussing C, Boerlin P, Daignault D, Dutil L, Reid-Smith RJ, Zhanel GG, Manges AR (2012). Chicken as reservoir for extraintestinal pathogenic *Escherichia coli* in humans. Canada Emerg Infect Dis.

[58] Araùjo S, Silva I, Tacão M, Patinha C, Alves A, Henrigques I (2017). Characterization of antibiotic resistant and pathogenic *Escherichia coli* in irrigation water and vegetables in household farms. Int J Food Microbiol.

[59] Giufrè M, Graziani C, Accogli M, Luzzi I, Busani L, Cerquetti M (2012). *Escherichia coli* of human and avian origin: detection of clonal groups associated with fluoroquinolone and multidrug resistance in Italy. J Antimicrob Chemother.

[60] Olsen RH, Chadfield MS, Christensen JP, Scheutz F, Christensen H, Bisgaard M (2011). Clonality and virulence traits of *Escherichia coli* associated with haemorrhagic septicaemia in turkeys. Avian Pathol.

[61] Haenni M, Beyrouthy R, Lupo A, Châtre P, Madec J-Y, Bonnet R (2017). Epidemic spread of *Escherichia coli* ST744 isolates carrying *mcr-3* and *bla*_CTX-M-55_ in cattle in France. J Antimicrob Chemother.

[62] El Garch F, Sauget M, Hocquet D, LeChaudee D, Woehrle F, Bertrand X. *mcr-1* is borne by highly diverse *Escherichia coli* isolates since 2004 in food-producing animals in Europe. Clin Microbiol Infect. 2017;23(51):e1–51 e4. 10.1016/j.cmi.2016.08.033 27615718

[63] Thiry D, Saulmont M, Takaki S, De Rauw K, Duprez JN, Iguchi A, Piérard D, Mainil JG. Enteropathogenic *Escherichia coli* O80:H2 in young calves with diarrhea, Belgium. Emerg Infect Dis. 2017;23:2093–5. 10.3201/eid2312.170450 PMC5708227 29148394

[64] Walk ST, Alm EW, Calhoun LM, Mladonicky JM, Whittam TS (2007). Genetic diversity and population structure of *Escherichia coli* isolated from freshwater beaches. Environ Microbiol.

[65] Dahms C, Hübner N-O, Kossow A, Mellmann A, Dittmann K, Kramer A. Occurrence of ESBL-producing *Escherichia coli* in livestock and farm workers in Mecklenburg-Western Pomerania, Germany. PLoS One. 2015;10:e0143326. 10.1371/journal.pone.0143326 PMC4659621 26606146

[66] Heidemann Olsen R, Christensen H, Kabell S, Bisgaard M (2018). Characterization of prevalent bacterial pathogens associated with pododermatitis in table egg layers. Avian Pathol..

[67] Abraham S, Kirkwood RN, Laird T, Saputra S, Mitchell T, Singh M, Linn B, Abraham RJ, Pang S, Gordon DM (2018). Dissemination and persistence of extended-spectrum cephalosporin-resistance encoding IncI1-*bla*_CTXM-1_ plasmid among *Escherichia coli* in pigs. ISME J.

[68] Mora A, López C, Herrera A, Viso S, Mamani R, Dhabi G, Alonso MP, Blanco M, Blanco JE, Blanco J (2012). Emerging avian pathogenic *Escherichia coli* strains belonging to clonal groups O111:H4-D-ST2085 and O111:H4-D-ST117 with high virulence-gene content and zoonotic potential. Vet Microbiol.

[69] Official Journal of the European Union. Commission notice. Guidelines for the prudent use of antimicrobials in veterinary medicine (2015). Available at https://ec.europa.eu/health/sites/health/files/antimicrobial_resistance/docs/2015_prudent_use_guidelines_en.pdf. Accessed 9 Jan 2019.

