# Supplementary material for: Phenotypic and genotypic characteristics of Escherichia coli with non-susceptibility to quinolones isolated from environmental samples on pig farms
Source: Porcine Health Manag. 2019 Feb 28;5:9. doi: 10.1186/s40813-019-0116-y (PMC6396500; doi:10.1186/s40813-019-0116-y)
Supplement: Supplementary file 1 — Table A1. Results of the MLST analysis for E. coli strains with new STs. (DOCX 26 kb) [file 40813_2019_116_MOESM1_ESM.docx]

***Supplementary Material***

Phenotypic and genotypic characteristics of *Escherichia coli* with non-susceptibility to quinolones isolated from environmental samples on pig farms

Patrick Kindle, Katrin Zurfluh, Magdalena Nüesch-Inderbinen^*^, Sereina von Ah, Xaver Sidler, Roger Stephan, Dolf Kümmerlen

*** Correspondence**: magdalena.nueesch-inderbinen@uzh.ch

Table S1. Results of the MLST analysis for *E. coli* strains with new STs.^a^

| **Strain**  **ID** | **PG** |  | **MLST alleles** | | | | | | |
| --- | --- | --- | --- | --- | --- | --- | --- | --- | --- |
|  |  |  | *adk* | *fumC* | *gyrB* | *icd* | *mdh* | *purA* | *recA* |
| 70 | B1 |  | 6 | 6 | 5 | 26 | 9 | 13 | 370 |
| 104 | B1 |  | 6 | 19 | 57 | 157 | 9 | 13 | 6 |
| 144 | E |  | 694 | 90 | 640 | 140 | 27 | 449 | 2 |
| 152 | E |  | 694 | 90 | 640 | 140 | 27 | 449 | 2 |
| 158 | A |  | 136 | 11 | 4 | 12 | 8 | 18 | 2 |
| 160 | A |  | 10 | 11 | 4 | 117 | 8 | 8 | 2 |
| 164 | A |  | 6 | 8 | 585 | 159 | 9 | 23 | 7 |

^a^The ST were not assigned numerical designations by the *E. coli* MLST database (http://mlst.warwick.ac.uk/mlst/dbs/Ecoli).

MLST, multilocus sequence type; PG, phylogenetic group; ST, sequence type.
